# Inhibition of the JAK-STAT Pathway in the Treatment of Psoriasis: A Review of the Literature

**DOI:** 10.3390/ijms25094681

**Published:** 2024-04-25

**Authors:** Andreea Roxana Furtunescu, Simona Roxana Georgescu, Mircea Tampa, Clara Matei

**Affiliations:** 1 Department of Dermatology, “Carol Davila” University of Medicine and Pharmacy, 020021 Bucharest, Romania; 2 Department of Dermatology, “Victor Babes” Clinical Hospital for Infectious Diseases, 030303 Bucharest, Romania

**Keywords:** psoriasis, psoriatic arthritis, Jak inhibitors, tofacitinib, deucravacitinib, upadacitinib

## Abstract

Psoriasis is a highly prevalent dermatological disease associated with an increased systemic inflammatory response. In addition, joint involvement is also present in around 20% of patients. Therefore, treatment modalities used in this condition should be simultaneously effective at improving skin manifestations, reducing inflammation, and addressing psoriatic arthritis when present. Twenty years ago, the introduction of biologic treatments for psoriasis was a turning point in the management of this condition, offering an effective and reasonably safe option for patients whose disease could not be adequately controlled with conventional therapies. At the moment, Janus Kinase inhibitors (JAKis) are a new class of promising molecules in the management of psoriasis. They are orally administered and can show benefits in patients who failed biologic therapy. We conducted a scoping review in order to identify randomized-controlled trials that investigated different JAKis in patients with plaque psoriasis and psoriatic arthritis, with an emphasis on molecules that have been approved by the European Medicines Agency and the Food and Drug Administration. The added value of this study is that it collected information about JAKis approved for two different indications, plaque psoriasis and psoriatic arthritis, in order to provide an integrated understanding of the range of effects that JAKis have on the whole spectrum of psoriasis manifestations.

## 1. Introduction

Psoriasis is one of the most prevalent chronic inflammatory diseases, affecting between 0.9% and 11.4% of the population of different geographic regions [1]. Forty to sixty million patients worldwide are estimated to have psoriasis [2,3,4].

From a dermatological point of view, psoriasis mainly presents with scaly, well-demarcated, erythematous plaques and nail dystrophy [5]. However, a significant proportion of psoriasis patients develop joint manifestations at some point in their disease course. Psoriatic arthritis (PsA) occurs in around 20% of psoriasis patients, with variations between studies, age groups, and geographic regions [6,7,8,9,10,11,12]. It is usually seronegative, asymmetric, and can be both axial and peripheric. Most often it involves the distal interphalangeal joints, sacro-iliac joints, and the spine. Radiographic changes also occur in PsA, as well as enthesitis, tenosynovitis, and dactylitis. PsA, especially active disease, is associated with an increased mortality rate [13].

Psoriasis and PsA are also associated with increased T-cell-derived inflammatory markers and an increased risk of coronary artery disease, atrial fibrillation, ischemic stroke, a hypercoagulable state, diabetes mellitus, and obesity [14,15,16,17,18,19]. 

In psoriasis, T-cells are overly activated, leading to increased levels of inflammatory cytokines such as tumor necrosis factor-α (TNF-α), interleukin (IL)-17A/C/F, IL-22, and interferon-γ (IFN-γ). The IL-23/IL-17 pathway is considered the main inflammatory pathway involved in psoriasis pathogenesis and it is the target of many systemic treatments for this disease. IL-17 leads to increased secretion of pro-inflammatory and antimicrobial molecules from immune cells and keratinocytes (G-CSF, GM-CSF. IL-6, CXCL1, CXCL2, CXCL5, CXCL8, IL-8, CCL20), which promote dysfunctional neoangiogenesis in the synovial membrane, recruitment of neutrophils into joint spaces, and endothelial inflammation. The activity of PsA and radiographic progression are associated with increased levels of IL-17^+^CD8^+^ T cells in the synovial fluid. Moreover, fibroblast-like synoviocyte proliferation leads to bone invasion and destruction. Similarly, modifications at the dermo-epidermal level include acanthosis, an increased cell turnover and the absence of granular layer, epidermal infiltrates of T-cells and neutrophils, and elongated and dilated capillaries in the dermal papillae. In addition, TNF-α plays a role in promoting inflammation and the migration of leukocytes, and IFN-γ promotes antigen presentation and the secretion of chemotactic factors such as CXCL9 and 10 and plays a part in establishing a positive feedback loop of inflammation [20,21,22,23]. 

Psoriasis and psoriatic arthritis have a significant impact on the quality of life. Patients have increased rates of anxiety, depression, fatigue, sexual dysfunction, and suicidal ideation and suffer from pain, itching, and sleep disturbances. The capacity to work is impacted as well. Patients with skin involvement, especially in visible areas, participate less in social activities [24,25,26,27,28,29,30].

Management of the psoriatic patient includes strategies for treating the dermatologic manifestations, as well as the musculoskeletal manifestations when they are present. Moreover, measures for improving the quality of life, decreasing systemic inflammation, and managing cardiovascular risk factors have to be taken. Therefore, moderate and severe forms should benefit from early systemic treatment in order to prevent disease progression and potential complications of the associated conditions [31,32,33,34,35].

The first biologic treatments for psoriasis that are still in use nowadays, TNF-α inhibitors, were introduced more than 20 years ago [36]. This was a turning point in the management of psoriasis, offering a reasonably safe therapeutic option for patients whose disease could not be adequately controlled with conventional treatments or who had contraindications to such treatments. Since then, other biologic therapies that have a more targeted mode of action have emerged. Classes of biologic treatments currently used in the treatment of psoriasis are TNF-α inhibitors, anti-IL-12/23, anti-IL-23, and anti-IL17 antibodies. Most of these drugs are human recombinant monoclonal antibodies. Exceptions to this rule are some TNF-α inhibitors: infliximab, which is a chimeric monoclonal antibody, certolizumab pegol, which is a humanized pegylated Fab fragment, and etanercept, which is a soluble fusion protein that combines two extracellular ligand-binding domains of the TNF p75 receptor with the Fc portion of human IgG1 [37,38]. 

However, in some patients, primary or secondary biologic treatment failure occurs. Currently, in such situations, a biologic switch is generally performed, but patients with previous biologic experience have lower response rates than biologic-naïve ones [39]. 

Moreover, because of their protein nature, biologic therapies are rapidly degraded in the gastrointestinal system and have to be administered parenterally, either by infusion or subcutaneous injection, which can be a source of distress for some patients [40,41,42].

At present, research on psoriasis treatment focuses on small molecules that can inhibit specific inflammatory pathways [43]. A new class of such small-molecule-drugs are Janus kinase inhibitors (JAKis. They act by blocking the evolutionarily conserved Janus kinase-signal transducer and activator of transcription (JAK-STAT) pathway, which is implicated in downstream signaling for cytokines using type I or II receptors. This makes JAKis appropriate for use in many immune-mediated inflammatory conditions, such as rheumatoid arthritis, lupus erythematosus, and graft-versus-host-disease [44,45,46,47]. 

Owing to their small molecular size (<500 kDa), JAKis can transit cellular membranes, exerting their effects at a cellular level. They are final drugs and do not require metabolization for activation. They are appropriate for oral, transcutaneous, or transmucosal administration. Therefore, JAKis can more easily be accepted by the patient and be employed for a wide range of indications [44,45,46,47,48].

Topical JAKi formulations have been used for treating atopic dermatitis, vitiligo, and alopecia areata. Topical JAKis reach high concentrations in the epidermis and upper dermis. After cutaneous application, the plasma concentrations are detectable, but low. However, the preparations should be applied on 20% or less of the skin surface, in a thin layer, in order to avoid side effects [46,49]. 

JAKs are non-receptor tyrosine kinases associated with the cytoplasmic domain of type I and II cytokine receptors. There are four types of JAK isoenzymes: JAK1, JAK2, JAK3, and tyrosine kinase 2 (TYK2) [45,46,47,48,50,51,52,53]. 

**Figure 1:**
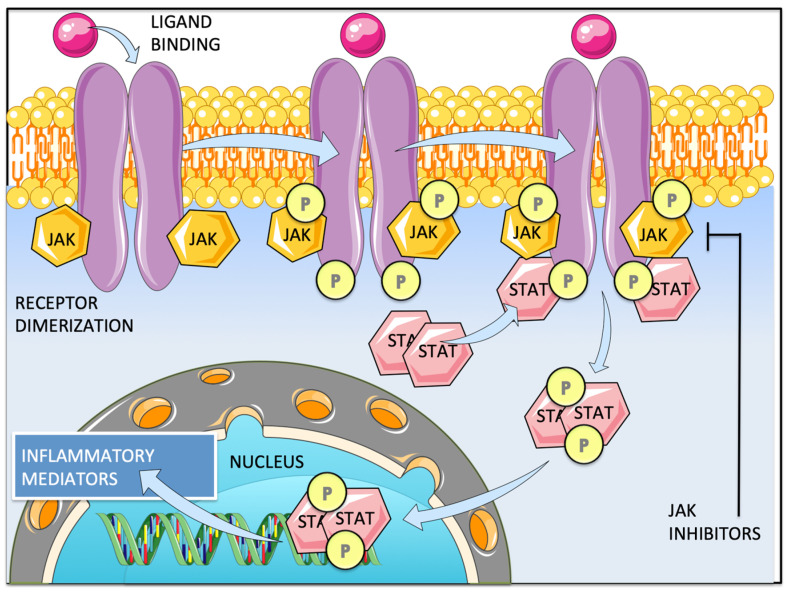
JAK/STAT signaling pathway. Cytokine binding induces receptor dimerization. Upon ligand binding, JAKs become activated and phosphorylate one another, as well as the intracellular domain of their receptor. This enables the recruitment, binding, and phosphorylation of cytoplasmic STATs, which consequently dimerize and translocate to the nucleus where they can then directly bind DNA, influence chromatin structure, and regulate gene expression of mediators of inflammation. The signaling pathways (cytokine/type of kinases/STATs) are detailed in Table 1.

Upon ligand binding, JAKs become activated and phosphorylate one another, as well as the intracellular domain of their receptor. This enables binding and phosphorylation of cytoplasmic STATs, which then dimerize and translocate to the nucleus where they can then directly bind DNA, influence chromatin structure, and regulate gene expression (Figure 1) [45,46,47,48,50,51,52,53].

Usually, two different types of JAKs are associated with the two chains of the receptor, forming heterodimers. JAK1/JAK3 heterodimers are implicated in signal transduction for cytokines that share a common γ-chain in their receptor, such as IL-2, IL-4, and IL-15. JAK1/JAK2 pairs are associated with receptors for IFN- γ and IL-6. JAK1/TYK2 pairs are implicated in the signaling pathways of type I interferons and IL-10 and JAK2/TYK2 in those of IL-12 and IL-23. One exception to this are the receptors for hematopoietic growth factors, which use JAK2 homodimers (Table 1) [45,46,47,48,50,51,52,53]. 

JAKis reversibly inhibit JAKs by binding at different catalytic or regulatory sites and preventing their ATP-mediated protein tyrosine kinase phosphorylation. The different substrates that bind JAKis are more or less conserved in the different isoenzymes of the JAK family. Drugs that bind to substrates that are more specific for a certain enzyme have a more targeted action. For instance, deucravacitinib binds to the regulatory pseudokinase domain of TYK2. This domain is less expressed across the other JAK isoenzymes, which means that deucravacitinib binds about 200 times easier to TYK2 than to JAK1 and 3. In contrast, tofacitinib binds to a highly conserved catalytic domain. It is therefore considered a pan-JAKi, even though it has some selectivity for JAK1 and 3 [54,58,62,63].

Many of the cytokines involved in psoriasis pathogenesis, such as IL-23 and IFN- γ, use JAKs for the downstream transduction of signal, which makes JAKis a promising therapeutic option for this condition [37]. The IL-23/IL-17 pathway, which is the main target of psoriasis treatment, uses JAKs for downstream transduction of signal [64].

IL-23 shares a common p40 subunit and a common IL-12Rβ1 receptor chain with IL-12. Upon binding to their receptor, IL-12 and IL-23 activate a JAK2-TYK2 heterodimer, which phosphorylates STAT3 and STAT4 and enables their transduction to the nucleus [53,65,66].

Il-12 is involved in immune responses by inducing IFN-γ production, promoting cell-mediated immunity, activating NK cells, and inducing T-cell proliferation. IL-12 also induces the differentiation of Th1 cells through a JAK-dependent mechanism and is implicated in the pathogenesis of inflammatory conditions with a strong Th1 response [66,67].

Il-23 and Th17 cells are involved in protection against bacterial and fungal infections [68]. While Th17 differentiation is induced by IL-6 and TGF-β, IL-23 is essential for Th17-cell function and production of key cytokines in psoriasis, such as IL-17A, IL-17F, IL-22, and TNF- α. IL-23 also increases IFN-gamma production, induces proliferation of memory T-cells, and promotes antigen presentation. Several cell types secrete increased levels of IL-17 in psoriasis: Th17 cells, γδ-, and αβ-T cells, neutrophils, mast cells, and NK cells [21,23,66,69]. IL-17 and TNF play a part in innate and adaptative immune responses as well [64]. 

The scope of this review Is to identify JAKis that have been evaluated for the treatment of plaque psoriasis and PsA. The novelty of our approach is that we looked into treatments used for both conditions at the same time. Almost a third of patients with psoriasis have articular disease and almost all patients with PsA also have cutaneous lesions [11,70,71]. Therefore, since two manifestations of this complex disease most often occur in the same patients, they should be tackled at the same time, even though they are managed by physicians from two different medical specialties—dermatology and rheumatology. We hope that our more patient-centered approach will be of value to both dermatologists and rheumatologists in fulfilling the needs of their patients. 

## 2. Methods

We conducted a scoping review in order to obtain a better understanding about the efficacy and safety of JAKis in the treatment of plaque psoriasis and psoriatic arthritis.

A PubMed search was performed on the 26 February 2024 for articles reporting data from clinical trials published since 2010 using the terms “JAK”, “JAK inhibitors”, “TYK2 inhibitors”, “tofacitinib”, “deucravacitinib”, “upadacitinib”, “abrocitinib”, “baricitinib”, “brepocitinib”, “filgotinib”, “peficitinib”, “ropsacitinib”, “ruxolitinib” “solcitinib”, “CP-690 550”, “BMS-986 165”, “ABT-494”, “PF-04965842”, “INCB-028050”, “ASP-015K”, “PF-06826647”, “INC-424”, and “psoria*”.

We selected phase 2 and 3, double-blinded, placebo-controlled, randomized clinical trials assessing the efficacy and safety of JAKis in the treatment of plaque psoriasis as psoriatic arthritis. We excluded phase 1 clinical trials because they were performed in a too-small number of patients to offer relevant information at this state of knowledge. We excluded RCTs evaluating the above-mentioned molecules in other inflammatory diseases. We excluded studies that did not have a control arm or that were not blinded. 

We performed searches for JAKi molecules in the FDA and the EMA databases in order to identify agents approved for the treatment of psoriasis or psoriatic arthritis and we concentrated on these molecules in our review because we think that in this way it brings greater value for the medical practice. 

We identified 9 phase 2 or 3, placebo-controlled, double blind, RCTs for tofacitinib, 4 for deucravacitinib, and 2 for upadacitinib. No topical JAKi is yet EMA- or FDA-approved for the treatment of psoriasis.

## 3. Results

We identified two orally administered JAKis, tofacitinib and upadacitinib, that have been approved by the Food and Drug Administration (FDA) and the European Medicines Agency (EMA) for PsA and one oral JAKi, deucravacitinib, for moderate-to-severe plaque psoriasis. No topical JAKi is yet approved for psoriasis. 

Tofacitinib has been evaluated in three phase 2 or 3 randomized clinical trials (RCTs) for PsA and in six such trials for moderate-to-severe plaque psoriasis. Deucravacitinib was evaluated in one phase 2 RCT for PsA and three phase 2 and 3 RCTs for moderate-to-severe plaque psoriasis. Upadacitinib was evaluated in two phase 3 RCTs for PsA.

Eight other oral JAKis not approved by the EMA or FDA for plaque psoriasis or psoriatic arthritis have been investigated in phase 2 and 3 RCTs for these indications. 

We also identified RCTs for three JAKis that have been applicated in topical formulations for mild-to-moderate psoriasis.

### 3.1. Efficacy

#### 3.1.1. Tofacitinib

Tofacitinib inhibits JAKs by competitively binding to a highly conserved ATP-binding site. It was originally developed as a JAK3 inhibitor, but it has some effect on all JAK isoenzymes. It inhibits signaling via JAK3 and JAK1 with a 5- to 100-fold selectivity over JAK2 and is even less active on TYK2 [72,73,74]. 

Tofacitinib is approved by the EMA and FDA for rheumatoid arthritis, psoriatic arthritis, ulcerative colitis, and ankylosing spondylitis in adults and for active polyarticular juvenile idiopathic arthritis in children from two years of age. For psoriatic arthritis EMA recommends a dose of 5 mg twice daily. The FDA advises to use tofacitinib at a dose of 5 mg twice daily or the extended-release preparation at a dose of 11 mg daily.

We identified one randomized, controlled, phase 2 trial and eight phase 3 trials assessing the clinical efficacy of tofacitinib in psoriasis and psoriatic arthritis [72,75,76,77,78,79,80,81] (Table 2). 

Other than the studies included in the table, one phase 2 study investigated the attenuation of psoriasis immune pathways with tofacitinib treatment [82].

- Moderate-to-Severe Plaque Psoriasis

**NCT00678210**. In 2012, tofacitinib was evaluated in a 12-week phase 2b dose-ranging trial (NCT00678210) in which 197 patients with moderate-to-severe plaque psoriasis were randomized to receive tofacitinib at a dose of 2 mg, 5 mg, or 15 mg twice daily or placebo for 12 weeks, followed by a 4-week surveillance period. The primary endpoint was the proportion of patients achieving a 75% reduction in their baseline Psoriasis Area Severity Index (PASI75) at week 12 [72]. At week 12, all the tofacitinib groups had significantly higher proportions of patients reaching PASI75 than placebo. Tofacitinib had a rapid effect, with a significant PASI75 response already being seen at week 4 and a significant PASI90 response at week 8. Higher doses led to better improvement of skin manifestations [72]. When the head and neck region, upper limbs, trunk, and lower limbs were analyzed separately, significant improvements occurred in all regions for all treatment groups versus placebo [83]. Dose-dependent decreases in C-reactive protein (CRP) levels were significant with the 5 and 15 mg twice daily doses of tofacitinib. This effect is probably due to decreased IL-6 activity following JAK-1 inhibition [84,85]. Patient-reported outcomes from this trial showed statistically significant and clinically meaningful improvements in Dermatology Life Quality Index (DLQI) scores, vitality, social function, and mental health [86]. The percentage of patients with a DLQI score of 0 or 1 (no effect on patient’s life) also increased starting with week 4 and up to week 12, in a dose-dependent fashion [72]. Moreover, tofacitinib provided a very rapid improvement of pruritus, with patients taking all doses of tofacitinib having significantly less pruritus than patients on placebo by day 6 (*p* < 0.05 at day 6 and *p* < 0.0001 by week 12). In the four follow-up weeks after the end of treatment, the intensity of pruritus worsened upon drug withdrawal. It was estimated that the effect of tofacitinib on pruritus was direct and not related to improvements in erythema, induration or scaling in a proportion of 70.2–80.5% [87]. Tofacitinib 5 and 15 mg twice daily also led to significantly decreased pain at weeks 4, 8, and 12 when compared to placebo [86]. More than 70% of participants in all treatment groups were “very satisfied” or “somewhat satisfied” with the treatment, especially in the 15 mg twice daily group. Also, the patients’ impression of their disease severity improved in a dose-dependent manner [86]. 

**NCT01186744**. This phase 3 trial that included a placebo-controlled treatment-withdrawal period and a re-exposure period was performed on 666 patients with moderate-to-severe plaque psoriasis. Patients were randomized in a 1:1 ratio to receive tofacitinib 5 mg or 10 mg twice daily for 24 weeks. Patients who did not achieve a PASI75 response and a Physician Global Assessment (PGA) score of 0 (clear) or 1 (almost clear) at week 24 were discontinued from the study. Patients who achieved the desired response (*n* = 291) were then randomized in a 3:1 ratio to either be switched to placebo or to continue treatment. The patients were switched to their initial tofacitinib dose at relapse or at week 40 and continued treatment up to week 56 [75]. The primary endpoints in the treatment-withdrawal period were the proportion of patients maintaining a PASI75 or a PGA response. The primary endpoints in the retreatment period were the proportion pf patients achieving a PASI75 or a PGA response among patients who relapsed. At week 24, 33.5% of patients in the 5 mg twice daily group and 55.2% in the 10 mg twice daily group achieved a PASI75 and a PGA response and were then re-randomized to enter the treatment-withdrawal period. Response rates for patients who were switched to placebo started to decline, while those in patients who continued treatment remained stable. Patients who were switched to placebo lost the PASI75 response in a median time of 8 weeks and relapsed after a median time of 16 weeks [75]. In the 16-week retreatment period, 36.8% and 61% of patients from the 5 mg and 10 mg twice daily groups who had gone through withdrawal regained a PASI75 response. Their response rate was lower at the end of the study than that of patients who had continued treatment in the treatment-withdrawal period. The DLQI scores of patients who entered withdrawal decreased as well but improved again after re-treatment and reached values similar to those in patients who had continued treatment [88].

**NCT01241591 (OPT Compare)**. This 12-week non-inferiority trial in patients with moderate-to-severe plaque psoriasis found that the 10 mg twice daily dose of tofacitinib (but not the 5 mg) was non-inferior to etanercept 50 mg twice weekly and superior to placebo. The rate of adverse events was similar for the two drugs [76]. Patients in all treatment groups also had clinically significant decreases in mean itch severity item score and clinically and statistically significant improvement in DLQI versus placebo. Improvements in patient-reported outcomes with tofacitinib were similar to etanercept [76,89]. Tofacitinib seems to have better results than placebo or etanercept in reducing pruritus. The itching sensation was significantly more reduced with tofacitinib as soon as the first day after starting treatment. Reductions of itching were significantly greater with tofacitinib 10 mg twice daily than with placebo or etanercept at all points between weeks 2–12 [76]. An extension study analyzed blood samples obtained at baseline and at week 4 from 266 of the participants in the tofacitinib 10 mg twice daily and etanercept arms of the study. One hundred and fifty-seven proteins involved in inflammation and in cardiovascular disease were dosed [90]. Changes in inflammatory and cardiovascular proteins at week 4 compared to baseline were correlated with response to treatment and with treatment with tofacitinib 10 mg twice daily. Post-treatment changes in IL-17A and IL-17C were correlated with the change in PASI score. Psoriasis and cardiovascular disease immune pathways were less active in responders with tofacitinib 10 mg twice daily and with etanercept than in non-responders. Both etanercept and tofacitinib treatments were associated with decreased levels of IL-6, CCL20, CXCL10, and IL-17A (only in responders). Some additional cardiovascular blood proteins only decreased in patients treated with tofacitinib (TNFR1, E-selectin, hK11, TRANCE, CHI3L1, IL-16, and matrix metalloproteinase-12) [90]. 

**NCT01276639 and NCT01276639 (OPT Pivotal 1 and 2).** Another paper [77] reported the results of two randomized, double-blind, phase 3 studies (OPT Pivotal 1, NCT01276639 and OPT Pivotal 2, NCT01309737) that evaluated tofacitinib against placebo in 901 and 960 patients with moderate-to-severe plaque psoriasis. Patients were randomized in a 2:2:1 ratio to receive tofacitinib at 5 mg or 10 mg twice daily or placebo, and the proportions of patients reaching PASI75 or a PGA score of 0 or 1 at week 16 were analyzed. At week 16, patients in the placebo arm were re-randomized to receive 5 or 10 mg of tofacitinib twice daily until week 52. Patients who did not show a PASI75 response or a PGA score of 0 or 1 at week 28 were withdrawn from the study. After 52 weeks, patients could either enter an extension open-label study or stop treatment and have a follow-up visit 2–4 weeks later. In both studies, tofacitinib performed significantly better than placebo for the primary outcomes. Patients on tofacitinib also had improved scores for PASI90 and quality of life when compared to placebo. The effect was better in the 10 mg twice daily group [77]. Patients with an increased body mass index (BMI) and patients with prior biologic experience had lower response rates [91]. Achieving a PASI50 response at week 8 was predictive of a PASI75 response at week 16 [92]. Tofacitinib also led to improved nail manifestations. The proportion of patients who achieved a reduction of at least 50% in their baseline Nail Psoriasis Severity Index (NAPSI50) in the two tofacitinib treatment arms became statistically significant against placebo at week 16, continued to rise until week 28, and was then stable up to week 52. In total, 32.8%, 44.2%, and 12% of patients in the tofacitinib 5 mg, 10 mg twice daily, and placebo groups achieved NAPSI50 at week 16 [93]. A subgroup analysis was performed for the Japanese patients in the OPT Pivotal 1 study [84]. There was a small number of only 58 Japanese patients enrolled in the study, out of which only 29 completed the study. The outcomes for this subgroup were similar to those seen in the global population of the study. Although direct comparisons between studies cannot be made, tofacitinib seemed to be slightly more efficacious in the East Asian population groups in this subgroup analysis and in other similar studies [78,81,94,95].

**NCT01815424**. This randomized, double-blind, phase 3, 52-week study of tofacitinib was conducted in Asian patients with moderate-to-severe psoriasis. A total of 266 patients were randomized to receive tofacitinib 5 or 10 mg twice daily or placebo that was switched to tofacitinib 5 mg or 10 mg twice daily at week 16. The patients were followed up to week 52. The primary endpoints of the study were the proportion of patients reaching PASI75 and obtaining a PGA score of 0 or 1 at week 16 [78]. In line with previous studies, tofacitinib met both primary endpoints for the two treatment doses at week 16 and the results were statistically significant. The median time to response was 8 weeks with 10 mg twice daily and 14–16 weeks for 5 mg twice daily. Patients in both treatment groups achieved significantly better results than placebo at week 12 for PASI90 and improvements in quality of life. Those in the 10 mg twice daily group also had a significant percentual change from baseline in NAPSI score at week 16. The majority of patients in the tofacitinib 5 mg and 10 mg twice daily groups maintained the PASI75 and PGA response achieved at week 16 through week 52.

**NCT01710046**. This randomized, placebo-controlled, double-blind, phase 2, 12-week study investigated the attenuation of immune pathways in patients with moderate-to-severe psoriasis. Twelve patients were randomized in a 3:1 ratio to receive tofacitinib 10 mg/day or placebo, and punch biopsies were taken from lesional and non-lesional skin at baseline and up to week 12. The study explored the relationship between efficacy endpoints and exploratory gene markers [82]. Patients treated with tofacitinib showed a rapid significant reduction in epidermal thickness and Ki67 expression levels beginning as early as day 1 after treatment and the normalization of the phenotype of lesional skin by week 12. There was evidence of a direct inhibition of the JAK/STAT pathway in keratinocytes, with levels of phosphorylated STAT1 and STAT3-positive nuclei declining as early as the first day of treatment. Keratinocytes also produced fewer inflammatory markers such as defensins and IL-36 by week 2. Genes that were upregulated in lesional skin started to decrease as early as week 2 and then returned to normal levels by week 12. Counts of CD11c+DC and CD3+ T-cells were reduced after 1 and 2 weeks of treatment and returned to levels of non-lesional skin at week 12. The p40 subunit that is common to IL-12 and IL-23 and plays a part in the activation of Th17 and Th1 cells was significantly reduced by week 2. Reductions were also seen in the levels of IFN-γ, IL-17, IL-19, CCL20, and molecules involved in the IFN-α and -β pathways. Tofacitinib does not directly inhibit IL-17, but it inhibits Th17 cell differentiation. IL-37, an anti-inflammatory cytokine, showed increased levels with treatment. Clinical and histologic improvement of psoriasis correlated with gene expression changes and with a reduction of IL-17 expression.

- Psoriatic Arthritis

**NCT01882439 (OPAL Beyond).** This 6-month, randomized, placebo-controlled, double-blind phase 3 trial evaluated the effects of tofacitinib in patients with psoriatic arthritis with a previous inadequate response to a TNF-α inhibitor. Three hundred and ninety-nine patients were randomized in a 2:2:2:1 ratio to receive tofacitinib at doses of 5 mg or 10 mg twice daily or placebo that was switched to tofacitinib 5 mg or 10 mg at 3 months. The primary endpoints were an ACR20 response and change from baseline in Health Assessment Questionnaire-Disability Index (HAQ-DI) at 3 months [79]. The primary endpoints were met, and results were significant for both doses of tofacitinib versus placebo at 3 months. Interestingly, at 3 and 6 months, the 5 mg twice daily groups had slightly better results than the 5 mg twice daily group. ACR20 responses were seen at a significantly higher rate with treatment than placebo early on, from week 2. ACR20 response and change from baseline in HAQ-DI continued to increase slightly until the 6 months of treatment. Both doses were also superior to placebo for ACR50 and ACR70 response rates. However, when PASI75 response was analyzed, only the 10 mg twice daily dose performed significantly better than placebo at 3 months. Patients also showed improvements in enthesitis, dactylitis, and physical function at 3 months, but results could not be tested for significance. Radiographic progression was not assessed in this study, but patients in both tofacitinib groups had more decreases in swollen and tender joint count, PGA of arthritis, Psoriatic Arthritis Response criteria (PsARC), and CRP levels when compared to placebo. Also, 23% of patients in the 5 mg twice daily group, 21% in the 10 mg twice daily group, and 15% in the placebo group met criteria for minimal disease activity (MDA) at 3 months. 

**NCT01877668 (OPAL Broaden).** This 12-month, randomized, placebo-controlled, double-blind phase 3 trial evaluated the effects of tofacitinib versus placebo and adalimumab in 422 patients with psoriatic arthritis with a previous inadequate response to a conventional synthetic disease-modifying antirheumatic drug (DMARD) (OPAL Broaden, NCT01877668). Patients were randomized in a 2:2:2:1:1 ratio to receive tofacitinib at doses of 5 mg or 10 mg twice daily, adalimumab 40 mg once every 2 weeks, or placebo that was switched to tofacitinib 5 mg or 10 mg twice daily at 3 months. The primary endpoints were an ACR20 response and change from baseline in HAQ-DI at 3 months [80]. The primary endpoints were met for both doses of tofacitinib against placebo, with a higher statistical significance for the 10 mg twice daily dose than for the 5 mg twice daily dose. Changes from baseline in ACR20 response rated and in HAQ-DI at 12 months were similar to those seen at 3 months. ACR50 and 70 responses were also significantly better with both doses of tofacitinib than placebo at 3 months. The effects of tofacitinib and adalimumab were similar. Also, patients had improvements in enthesitis, dactylitis, PASI75, and patient-reported outcomes [96]. Minimal radiographic progression was seen in all treatment groups at 12 months, with 95.9% and 94.9% of patients in the 5 mg and 10 mg tofacitinib groups having radiologic non-progression defined as a modified Sharp score of ≤0.5. Slightly more patients in the adalimumab group had radiographic non-progression (97.9%). An elevated CRP at baseline was associated with greater structural progression. CRP levels decreased in all treatment groups, with a slower onset of the reduction in the tofacitinib 5 mg group [97]. In the OPAL Beyond and OPAL Broaden, patient-reported outcomes such as pain, fatigue, physical function, and patients’ own assessment of their disease improved up to 3 months and remained stable until the end of the study. Improvements with tofacitinib were similar to those seen with adalimumab. The improvements in quality of life occurred at the same time as the improvement of dermatologic manifestations [96,98]. 

A post-hoc analysis of patients in the OPAL Beyond and OPAL Broaden trials was made taking into account the BMI of patients. Tofacitinib had better treatment outcomes than placebo for all BMI categories. Patients with a BMI ≥35 had lower response rated overall, especially in the tofacitinib 10 mg twice daily group. Baseline BMI seemed to be a predictor for ACR50/70, minimal disease activity (MDA), and HAQ-DI response rates at 3 months in the group receiving 5 mg twice daily. Safety profiles seemed similar to the global study population, except for more frequent elevation in liver enzymes in the group receiving tofacitinib 5 mg twice daily [99].

**NCT03486457.** A randomized, controlled, double-blind, phase 3, 6-month trial (NCT03486457) was conducted in China in patients with psoriatic arthritis that had prior inadequate response to conventional DMARDs [81]. The 204 patients who participated in the study were randomized in a 2:1 ratio to receive either tofacitinib 5 mg twice daily or placebo that was switched to tofacitinib 5 mg twice daily after 3 months. The primary endpoint was the proportion of patients achieving ACR50 at 3 months. At 3 months, 38.2% of patients in the tofacitinib group achieved ACR50 as compared to 5.9% with placebo (*p* < 0.0001). Better improvements with tofacitinib were seen from the first month. Improvements in ACR continued until the sixth month in the group receiving continuous tofacitinib. PASI75 improvement was also seen more with tofacitinib than placebo at months 1 and 3. There were also improvements in the quality of life, physical and mental health scores, pain, dactylitis, and enthesitis. CRP levels were also reduced with treatment versus placebo at 3 months. Radiographic progression was not assessed in this study. However, when compared to placebo, more patients in the tofacitinib 5 mg twice daily group had improvements in HAD-QI and PGA, as well as reductions in the number of swollen and tender joints. More patients achieved MDA with tofacitinib versus placebo (32.4% vs. 5.9%). 

#### 3.1.2. Deucravacitinib

Deucravacitinib is approved by the EMA and the FDA in the treatment of moderate-to-severe plaque psoriasis in patients who are eligible for systemic therapy. It is administered at a dose of 6 mg once daily.

Deucravacitinib is a highly selective oral TYK2 inhibitor with a more targeted mode of action and a superior safety profile. Normally, when binding to their receptors, IL-12 or IL-23 induce the phosphorylation of JAK2 and TYK2 and further signal transduction. Deucravacitinib inhibits this pathway [62,100,101].

Deucravacitinib has a 200-fold greater selectivity for TYK2 than for JAK1 and JAK3 and a 3000-fold greater selectivity for TYK2 than for JAK2 [58,101]. This is because, as opposed to previously developed JAKis, deucravacitinib binds to a catalytically inactive pseudokinase regulatory domain that is more specific for TYK2 than for JAK1-3. When it binds to TYK2, deucravacitinib inhibits downstream signaling by inducing conformational changes that impede the crucial interaction between the catalytic and pseudokinase domain [100]. 

We identified three randomized controlled phase 2 or 3 trials with published results that evaluate the efficacy of deucravacitinib in plaque psoriasis and one in PsA (Table 3).

- Moderate-to-Severe Plaque Psoriasis

**NCT02931838.** This phase 2, double-blinded, randomized trial was conducted in 267 patients with moderate-to-severe psoriasis, who received deucravacitinib at doses of 3 mg every other day, 3 mg daily, 3 mg twice daily, 6 mg twice daily, 12 mg daily, or placebo for 12 weeks. The patients were followed up for a 30-day period after the end of treatment. The primary endpoint evaluated the proportion of patients reaching PASI75 at week 12 [102]. Patients who received deucravacitinib at doses of 3 mg per day and higher performed significantly better than patients in the placebo group. The treatment proved to be effective early on, at day 15, and its effects were still visible after the 30-day follow-up period, even though the response was starting to wane. The proportion of patients who attained PASI75 and who had not previously used a biologic agent was between 12 and 81% in all treatment groups and between 5–67% in patients who had already used a biologic agent. The treatment also improved the quality of life in a dose-dependent manner for groups of 3 mg twice a day or higher. 

**NCT03624127 (POETYK PSO-1).** This randomized, controlled, phase 3, 52-week trial evaluated the efficacy of deucravacitinib against placebo and apremilast. Patients (*n* = 666) with moderate-to-severe psoriasis were randomized in a 2:1:1 ratio to receive 6 mg of deucravacitinib per day, placebo, or 30 mg of apremilast twice daily for 16 weeks. After week 16, the patients from the placebo group were switched to deucravacitinib. After week 24, patients from the apremilast group who had not reached PASI50 were also switched to deucravacitinib. The primary endpoints were the response rate of PASI75 and PGA of 0 or 1 of deucravacitinib versus placebo at week 16 [103]. Primary endpoints were met, with a significantly higher proportion of patients showing the desired responses with deucravacitinib (58.4%/53.6% for PASI75/PGA response) than placebo (12.7%/7.2%). Deucravacitinib also performed significantly better than apremilast at weeks 16 and 24. PASI response to deucravacitinib increased until week 24 and was then maintained up to week 52. Deucravacitinib showed better results than placebo or apremilast in treating scalp and nail psoriasis. Also, a statistically significant higher proportion of patients in the deucravacitinib group had a DLQI of 0 or 1 at week 16 than placebo or apremilast (41% vs. 10.6% vs. 28.6%). 

**NCT04036435 (POETYK PSO-2).** POETYK PSO-2 was a randomized, double-blinded, phase 3 trial in which 1020 patients with moderate to severe plaque psoriasis were randomized in a 2:1:1 ratio to receive deucravacitinib 6 mg per day, placebo, or apremilast 30 mg twice daily. Patients who were initially randomized to the deucravacitinib and who obtained a PASI 75 response until week 24 were then randomized in a 1:1 ratio to either continue deucravacitinib or switch to placebo and enter a withdrawal period. Patients who were initially randomized to placebo crossed over to deucravacitinib at week 16. Patients who were initially randomized to the apremilast and who obtained a PASI 75 response before week 24 were switched to placebo and those who had not yet obtained a PASI75 response to deucravacitinib. After 52 weeks, patients could enter the open-label extension trial POETYK PSO-LTE. The primary outcomes of the study were reaching PASI75 and a PGA score of 0 or 1 with an at least two-point improvement from baseline at week 16 for deucravacitinib as compared to placebo [104]. The primary outcomes were met. Moreover, deucravacitinib performed better than apremilast at weeks 16 and 24. Response rates with deucravacitinib increased up to week 24 and then 80.4% of patients remained stable until 52 weeks. In patients who were switched to placebo, the response decreased until week 40 and then remained relatively stable, with 31.1% of patients conserving the PASI75 response at 52 weeks. Patients who were switched to placebo lost the PASI75 response after a median time of 85 days. Deucravacitinib had significantly better results than placebo and apremilast in scalp psoriasis and at improving the quality of life (PSSD and DLQI) and led to more improvement of nail psoriasis than placebo in the small number of affected patients in the study.

Of the patients that participated in the two POETYK trials, 1221 entered the long-term extension open-label trial POETYK LTE (NCT04036435), in which all participants received deucravacitinib at a dose of 6 mg daily. Data published so far showed that patients who continued deucravacitinib treatment maintained their response rates at 2 years [106].

- Psoriatic Arthritis

**NCT03881059**. Deucravacitinib was also evaluated in a randomized, double-blind, phase 2 trial for psoriatic arthritis (NCT03881059). The 203 patients were randomized in a 1:1:1 ratio to placebo or deucravacitinib 6 mg or 12 mg daily. The primary endpoint was ACR20 response at week 16 [105]. The ACR20 response at week 16 was significantly higher with deucravacitinib versus placebo (31.8%) and better with 12 mg (62.7%) versus 6 mg (52.9%) of deucravacitinib. Improvements in ACR20 occurred early, being already visible at week 8. Patients who had already had treatment with a TNFα inhibitor also had higher response rates with deucravacitinib. Radiographic progression was not assessed in this study, but the HAQ-DI score was significantly reduced in patients in the deucravacitinib groups when compared to placebo at week 16, with improvements already apparent from week 4. Significantly better results were seen with deucravacitinib vs. placebo with respect to obtaining a PASI75 response, resolution of dactylitis and enthesitis, quality of life, and patient well-being. 

#### 3.1.3. Upadacitinib

Upadacitinib is approved in adults for the treatment of rheumatoid arthritis, psoriatic arthritis, ulcerative colitis, Crohn’s disease, ankylosing spondylitis and non-radiographic axial spondyloarthritis. It is also approved in adults and children over 12 years old with atopic dermatitis. The recommended dose for psoriatic arthritis is 15 mg daily. 

Similar to tofacitinib, upadacitinib reversibly binds to the catalytic domain of JAK enzymes through ATP competitivity. Upadacitinib is an oral reversible JAKi that has increased selectivity for JAK1 over JAK2, JAK3, and TYK2 [58,107].

Two randomized, placebo-controlled, phase 3 trials assessed its efficacy in PsA (Table 4).

- Psoriatic Arthritis

**NCT03104400 (SELECT-PsA 1).** In the SELECT-PsA 1 clinical trial, 1704 patients with PsA who had previously had inadequate response with conventional synthetic DMARDs were randomized to receive upadacitinib at a dose of 15 mg or 30 mg per day, placebo, or adalimumab at a dose of 40 mg every two weeks [108]. Patients in the placebo group were randomly switched to upadacitinib 15 mg or 30 mg at week 24. The study continued until week 56, at which point patients could choose to continue in an open-label extension study [110]. The primary endpoint was an ACR20 response at week 12 with upadacitinib versus placebo. The primary endpoint was met, and both upadacitinib doses showed non-inferiority to adalimumab at week 12. Moreover, the 30 mg dose was superior to adalimumab. The patients who were switched from placebo showed responses similar to their respective upadacitinib groups at week 56 for most aspects, showing a rapid improvement. At week 24, both doses of upadacitinib were already better than placebo at inhibiting the radiographic progression of the disease, improving the quality of life, decreasing fatigue and pain, and improving enthesitis. The effects were maintained through week 56. At 56 weeks, the improvement of enthesitis and dactylitis was similar for upadacitinib and adalimumab. The proportion of patients with no radiographic progression at week 56 evaluated by the modified Sharp–van der Heijde score was 97% for the upadacitinib 15 mg group, 96% for the 30 mg group, and 94% for adalimumab. The results for both doses of upadacitinib were significantly better than those for placebo (89%, calculated by linear extrapolation). More patients in the 30 mg upadacitinib group reached MDA than with adalimumab [108]. Concerning the cutaneous manifestations, when compared to placebo at week 24, both doses of upadacitinib led to a significantly higher proportion of patients that reached PASI75 or a PGA of 0 or 1 with a decrease of at least two points from baseline. Skin outcomes were maintained through week 56. Patients who had initially been randomized to placebo and were later switched to upadacitinib had responses similar to those who had been on the treatment from the beginning at week 56. Results published from the open-label extension study showed that outcome measures were maintained at 2 years [110]. 

**NCT03104374 (SELECT-PsA 2)**. In the SELECT-PsA 2 56-week, randomized, controlled, double-blind, clinical trial, 642 patients with PsA refractory to biologic therapy were randomized in a 2:2:1:1 ratio to once per day 15 mg or 30 mg upadacitinib or placebo for the first 24 weeks, then switched to 15 mg or 30 mg upadacitinib. The primary endpoint was an ACR20 response at week 12 with upadacitinib versus placebo. After 56 weeks, patients could enter an open-label study until week 152 [109,111]. Both doses of upadacitinib proved their superiority to placebo in achieving ACR20 at week 12 and the 30 mg. The 30 mg once daily dose had better results than the 15 mg dose (63.8% with 30 mg, 56.9% with 15 mg, and 24.1% with placebo, *p* < 0.001). The ACR20 response rates rose until week 12 for participants in the 30 mg group and until week 20 for the 15 mg group, then were conserved until week 56. At this point, similar ACR responses were seen with the 15 mg and the 30 mg doses. Patients who had been switched from placebo had similar results to those who had taken upadacitinib from the beginning. Response rates for patients who had already failed more than one biologic treatment were similar to those of the global study population. Upadacitinib showed efficacy as a monotherapy or in combination with conventional synthetic DMARDs. MDA was achieved at significantly higher rates compared to placebo at week 24: 25.1% for patients in the 15 mg group and 28.9% for the 30 mg group. At week 56 more patients who had been on upadacitinib from the beginning achieved MDA than those who had been switched from placebo. Improvement in skin manifestations assessed through PASI75/90 and PGA response rates was also seen in significantly higher proportions in patients treated with tofacitinib at weeks 12, 24, and 56 when compared to placebo. Better responses were seen with the higher dose. Patients in both treatment groups had significantly better outcomes for resolution of dactylitis or enthesitis, physical function, quality of life, and fatigue as well [109,111]. However, the SELECT-PsA 2 trial did not assess the effects of upadacitinib on radiographic progression [109,111]. A post-hoc analysis of patient-reported outcomes from the SELECT-PsA 1 and 2 studies showed the efficacy of upadacitinib at a dose of 15 mg per day at improving the symptoms of patients with PsA and axial involvement. The Bath Ankylosing Spondylitis Disease Activity Index (BASDAI) and Ankylosing Spondylitis Disease Activity Score (ASDAS) were compared at 12, 24, and 56 weeks for placebo, 15 mg of upadacitinib and adalimumab. Upadacitinib at a dose of 15 mg daily showed significantly better results than placebo at week 12 and 24. At week 12, adalimumab performed slightly better than upadacitinib, but at weeks 24 and 56, there was a numerically greater reduction in BASDAI and ASDAS with upadacitinib 15 mg than with adalimumab [112]. At 152 weeks, efficacy endpoints for musculoskeletal disease, cutaneous manifestations and patient-reported outcome were conserved, with few differences between the 15 mg and 30 mg dose [113].

#### 3.1.4. Other oral JAKis

Even though they have not received approbation for this indication, other JAKis have been evaluated in clinical trials for psoriasis or PsA. 

**Abrocitinib** showed PASI75 responses in up to 60% of patients in a phase 2 clinical study. However, the sponsor discontinued support for the study and the enrollment period was not completed as planned. The drug is now approved for the treatment of atopic dermatitis [85].

**Baricitinib** is a JAK1/JAK2 inhibitor approved for rheumatoid arthritis, atopic dermatitis, alopecia areata, and juvenile idiopathic arthritis. In a phase 2 trial, it showed PASI75 responses in 43% of patients treated with 8 mg per day and 54% of patients treated with 10 mg per day, as opposed to only 17% in the placebo group [114].

**Brepocitinib** is a TYK2/JAK1 inhibitor that has not yet been approved by the EMA or the FDA. A phase 2a trial in patients with moderate-to-severe psoriasis showed significant reductions in PASI score at week 12 when compared to placebo [115]. Brepocitinib also showed a significantly improved ACR20 response at week 16 when compared to placebo in a phase 2b clinical trial for PsA, with conservation of the response up to week 52 [116]. The drug was generally well-tolerated in the two studies. 

**Filgotinib** is only approved by the EMA for rheumatoid arthritis and ulcerative colitis. Patients with active PsA treated with filgotinib in a phase 2 trial achieved significant improvements in ACR response, peripheral arthritis, psoriasis, enthesitis, and patient-reported outcomes [117].

**Itacitinib** is a JAK1 inhibitor that is being investigated for the treatment of acute graft-versus-host disease [118]. In a phase 2 study for plaque psoriasis, it only led to significant improvements at a dose of 600 mg daily [119].

**Peficitinib** is a pan-JAK inhibitor that is approved for the treatment of rheumatoid arthritis in Korea, Japan, and Taiwan that also induced significant changes in PASI score versus placebo at 6 weeks [120,121].

**Ropsacitinib** is a selective TYK2 inhibitor that was associated with a significant PASI90 response at week 16 versus placebo (*p* < 0.0001), which increased up to week 40 [122]. 

**Solcitinib** is a selective JAK1 inhibitor for which no clinical trials are ongoing at the moment. It showed improved PASI75 responses at 12 weeks in a phase 2a study in patients with psoriasis in 2016 [123].

#### 3.1.5. Topical JAKis

**Brepocitinib.** Topical formulations with different brepocitinib concentrations applied once or twice daily were evaluated against vehicle in a phase 2b double-blind trial. A total of 344 patients were randomized and received treatment in the first 12-week stage of the study. However, brepocitinib failed to induce statistically significant improvements when compared to placebo. The treatment was generally well tolerated. There was one patient in the study who developed herpes zoster and herpes simplex lesions a few days apart, in areas not involved in psoriasis plaques [124]. 

**Ruxolitinib.** After demonstrating similar efficacy to calcipotriol and betamethasone formulations in a small double-blinded trial [125], ruxolitinib was evaluated in an unblinded study. Ruxolitinib showed improvements in lesion area and severity when compared to non-treated sites. It also led to improvements in calculated PGA scores. The formulations were generally well-tolerated. Two patients reported local reactions, stinging and hypoesthesia, one patient developed mild leukopenia and another one mild reticulocytosis [126]. 

**Tofacitinib**. Two topical formulations of tofacitinib 2% ointment were tested against vehicle in a phase 2a study in patients with mild-to-moderate psoriasis. One of the ointments achieved a statistically significant TPSS reduction when compared to vehicle at week 4. Treatment was generally well tolerated. A few patients reported local erythema and a stinging or burning sensation [127]. A phase 2b study that investigated two concentrations of a tofacitinib ointment (1% or 2%) against placebo in 430 patients with mild-to-moderate psoriasis found a significant improvement against vehicle for the 2% formulation, but no significant differences from vehicle at week 12 [128]. 

### 3.2. Safety

#### 3.2.1. Tofacitinib and Upadacitinib

Considering the mode of action of tofacitinib and upadacitinib, major events of concern are serious infections, major adverse cardiovascular events (MACE), venous thromboembolism (VTE) events, and malignancy. 

Some of the RCTs reported in our review were continued by open-label long-term extension studies that provide more reliable information about the occurrence of such rare adverse events. Patients with psoriasis treated with tofacitinib in OPT Pivotal 1 and 2 could choose to continue in an open-label extension study, which provided safety data for up to 66 months of treatment [129]. The OPAL Balance open-label extension study provided safety data at 48 months for the use of tofacitinib in PsA [130]. PsA patients treated with upadacitinib in the SELECT-PsA1 and 2 studies could continue in open-label extension studies, for which data were published after 2 and 3 years, respectively [110,113]. The extension study of SELECT-PsA1 is still ongoing and is expected to provide further data at the end of 5 years of treatment. This study also includes an adalimumab-treated patient group, providing an interesting comparison between biologic treatments and JAKis, even though the study design does not provide statistical power for such a comparison [110]. 

The most commonly reported adverse events with both drugs were nasopharyngitis and upper respiratory tract infections [75,76,77,78,79,80,81,108,109,110,113,129,130]. More serious infections were reported with tofacitinib and upadacitinib than placebo and with upadacitinib than adalimumab, especially with higher doses [75,76,77,78,79,80,81,108,109]. Herpes zoster seems to occur more frequently in patients taking tofacitinib and upadacitinib when compared to placebo, although most cases were mild to moderate and occurred in patients who had not had previous herpes zoster episodes and were not vaccinated [109,113,129,130]. More herpes zoster cases occurred with upadacitinib, especially at the 30 mg dose, than with adalimumab [110,113]. Some cases of disseminated herpes zoster infections occurred [110,113,129,130]. VZV reactivation seems to occur more frequently in East Asian patients [78,81,94,95]. A few cases of opportunistic infections were seen, such as urethral, oropharyngeal, and tracheal candidiasis, opportunistic herpes zoster, and bronchopulmonary aspergillosis [80,108,109].

Regarding laboratory studies, moderate, dose-dependent decreases in hemoglobin values were seen with both tofacitinib and upadacitinib. There were a few reports of anemia with a hemoglobin level of less than 10 g/dL. With tofacitinib, there was an initial decrease in neutrophil counts in the first month of treatment, which then started to resolve despite continuation of treatment. On the contrary, lymphocyte levels rose at the beginning of treatment, but then started to decrease [54]. No such variations were seen with upadacitinib [109]. However, a few cases of neutropenia and lymphopenia were reported with both drugs. Hematological parameters normalized upon drug discontinuation. 

Dose-dependent increases in LDL and HDL cholesterol values occurred with both drugs, without significant modification in the LDL/HDL ratio. Cholesterol levels rose in the first 1–3 months of treatment and then stabilized [72,76,80]. Cholesterol levels returned to baseline levels after the interruption of treatment and rose again upon rechallenge [75].

CPK elevations were seen with both drugs, even though these were usually moderate and only a few cases were associated with myalgia or were high enough to prompt treatment discontinuation. No cases of rhabdomyolysis were reported [75,76,77,78,79,80,81,108,109,110,113]. 

Liver transaminase levels increased fairly commonly with treatment, but the elevations were usually mild and did not fulfill Hy’s law criteria. There were, however, a few reports of values of more than three times the upper limit of the normal range [75,76,77,78,79,80,81,108,109,110,113].

Malignancy, MACE, and VTE are effects of special interest since patients with psoriasis or PsA already have a higher cardiovascular and malignancy risk than the general population. Such events were reported sporadically in clinical trials for both drugs, but their incidence rate was not obviously higher than that of the general population and no specific type of cancer was associated with JAKi use [75,76,77,78,79,80,81,108,109]. Analyses of longer-term data showed similar incidence rates of MACE, VTE, and malignancy in patients with PsA and plaque psoriasis treated with JAKis or biologic drugs [110,113,129,130]. The risk of cardiovascular events, but also of malignancy, is increased in patients who have an increased cardiovascular risk at baseline, a history of prior cardiovascular events, diabetes, high blood pressure, and obesity [131,132,133]. More data from long-term real-world studies are needed in order to establish a more a reliable safety profile. 

There were more reported adverse events and serious adverse events with tofacitinib and upadacitinib than with placebo [75,76,77,78,79,80,81,108,109]. More events were seen with the 30 mg than with the 15 mg dose of upadacitinib, but the 5 and 10 mg twice daily doses of tofacitinib seemed to have similar safety profiles [110,113,129,130]. However, both drugs were generally well tolerated and showed a similar safety profile with biologic or conventional DMARDs already in use in psoriasis and PsA [110,113,129,130,131,132,133,134]. 

#### 3.2.2. Deucravacitinib

Deucravacitinib has increased selectivity in targeting TYK2 over other JAKs, having a more targeted action that confers a superior safety profile, with less expected hematologic and thromboembolic adverse events [101,135]. Over 90% of adverse events were mild and moderate in severity [106]. The rate of adverse events and serious adverse events seen with deucravacitinib is similar to apremilast [103,104].

Similar to other JAKis, the most commonly seen adverse events with deucravacitinib were nasopharyngitis and upper respiratory tract infections. When compared to apremilast, deucravacitinib was associated with fewer cases of headache, diarrhea, and nausea and more cases of nasopharyngitis and upper respiratory tract infections [103,104].

Unlike the previously discussed JAKis, no significant changes from baseline in blood count parameters, levels of liver enzymes, cholesterol, creatinine, or CPK occurred in groups treated with deucravacitinib. Lymphopenia, anemia, thrombocytopenia, and neutropenia were not associated with deucravacitinib treatment [102,103,104,105,106].

However, more cases of acne and folliculitis seem to occur in patients treated with deucravacitinib, even though incidence rates are not high and cases are not serious. The mechanism remains unknown, but could be related to alterations of the skin microbiome induced by the changes in immune response with deucravacitinib treatment [106]. 

Data published after 2 years from the ongoing long-term extension open-label trial POETYK LTE (NCT04036435) provide the most relevant information available so far regarding the risk of malignancy, MACE, and VTE events in patients with psoriasis treated with deucravacitinib. The rate of MACE and VTE was similar to available data from patients receiving other systemic treatments for psoriasis, including biologic therapies. Nine patients developed MACE, and three developed VTE events. New cases of malignancies, including non-melanoma skin cancers, occurred at rates similar to the general population, and there was no observed association with any particular type of cancer [106].

## 4. Discussion

Tofacitinib, upadacitinib, and deucravacitinib showed efficacy comparable with that of other existing treatment modalities for psoriasis and PsA. They also demonstrated an acceptable safety profile [72,75,76,77,78,79,80,102,103,104,105,109,136]. 

Greater efficacy was observed with higher JAKi doses for most outcomes. However, the use of higher doses entails a loss of selectivity of tofacitinib and upadacitinib. This means that JAK2 is also inhibited, and blood count parameter levels are more affected [72,75,76,77,78,79,80,109,136]. 

Cholesterol levels were shown to increase in a dose-dependent manner with tofacitinib and upadacitinib. Even though absolute levels did not increase very much, levels of blood lipids should be carefully monitored, since psoriasis patients already have an increased cardiovascular risk [72,75,76,77,78,79,80,109,136].

There are some laboratory parameters that can be measured in order to highlight the effect of JAKis on certain pathways. Decreases in reticulocyte, thrombocyte, and neutrophil counts are indicators of the JAK2-mediated inhibition of erythropoietin and thrombopoietin signaling. Interferon-γ (IFN-γ) induced protein 10 (IP-10) levels decrease when JAK1 inhibition leads to further inhibition of the IFN signaling pathway. Decreased high-sensitivity C reactive protein (hsCRP) levels are an indicator of the inhibition of the IL-6 pathway, following JAK1 inhibition [85].

Data from the existing studies show a significant amelioration of pruritus with JAKis, that occurs even from the first days of treatment. JAKis seem to have better results in this area than TNF-α inhibitors or apremilast [76,103,104]. This could bring a valuable addition to psoriasis treatment, since for many patients, pruritus is the most bothersome manifestation of the disease [137].

A valuable place in psoriasis therapy for JAKis derives from the fact that they are efficient in difficult-to-treat patient populations, such as patients who have failed biologic therapy. Efficacy upon rechallenge with the same JAKi has also been demonstrated [75].

The introduction of the highly selective TYK2 inhibitor, deucravacitinib, was a great stepping stone for the treatment of psoriasis, providing effective treatment with an improved safety profile [102,103,104,105]. Without any doubt, other selective JAKis will be developed. 

Infections occur at a higher rate in patients treated with JAKis. However, the most commonly reported infectious events were mild to moderate nasopharyngitis and upper respiratory tract infections, and serious and opportunistic infections remained rare. In most studies, the incidence of infectious events was higher when higher medication doses were used. There is an increased incidence of herpes zoster with JAKi treatment, but most cases are mild to moderate and few cases of disseminated infection were reported [110,113,129,130].

Due to their less selective mechanism of action, tofacitinib and upadacitinib are associated with more adverse events such as anemia, neutropenia, lymphopenia, dyslipidemia, and elevation of transaminases [72,75,76,77,78,79,80,109,136]. 

Moreover, JAKis have the potential to be used in topical formulations because they do not need to be activated in another organ in order to exert their effects and because of their small molecule size. This could offer new therapeutic options for the milder cases of psoriasis, especially for special sites, in which corticosteroids often induce side effects. However, no topical formulation tested so far showed satisfactory enough results [125,126]. 

In 2015, in a complete response letter, the FDA refused the approval of Pfizer’s application for tofacitinib in the treatment of psoriasis, requesting more safety data [138]. Tofacitinib was, however, approved for the treatment of PsA in 2017. There is an FDA black box warning for increased risk of infection, mortality, malignancy, MACE, and VTE with JAKis. On 23 January 2023, EMA’s human medicines committee endorsed a series of recommendations that are meant to reduce the potential risk of serious events associated with JAKi use. The EMA recommends to only use JAKis if another treatment option is not possible in patients over 65 years old, smokers, and patients at high risk of major cardiovascular events. Caution and dose reductions are advised in patients with venous thromboembolism, cancer, or major cardiovascular problems [139]. 

Experience with JAKis is still limited, especially regarding long-term use. Therefore, clinicians should keep an eye on papers reporting new side-effects of these drugs. Long-term, real-world evidence is needed in order to fully understand the safety profile of JAKis, especially concerning rare events. Several open-label studies for JAKis are ongoing, and results from these studies will help to better understand the real safety profile of this new class of drugs. Even though in psoriasis and PsA patients treated with JAKis, the risk of major cardiovascular events and malignancy seems similar to the global population with these diseases [131,132,133], data from a 4-year open label study in rheumatoid arthritis showed increased rates of major cardiovascular events and malignancy with tofacitinib compared to TNF-α inhibitors [140]. 

## 5. Conclusions

Oral JAKis are a therapeutic option that should be taken into consideration for the treatment of moderate-to-severe psoriasis and psoriatic arthritis. As shown by studies in patients who had already failed treatment with conventional synthetic or biologic DMARDs, JAKis are effective even in difficult-to-treat forms of psoriasis.

Tofacitinib, upadacitinib and deucravacitinib have shown results similar to those of well-established psoriasis and PsA treatments etanercept, adalimumab and apremilast.

Even though molecules are approved either for the indication of plaque psoriasis or for PsA, studies have shown that they are efficient in tackling both the dermatological and the rheumatological manifestations of disease. This is very useful in clinical practice since many psoriasis patients also have PsA manifestations at the time of presentation.

JAKis have an acceptable safety profile, similar to that of adalimumab or etanercept. They were, however, associated with an increased risk of herpes zoster, serious infection, and modifications in laboratory parameters, especially when used at higher doses. Owing to its high specificity for TYK2, deucravacitinib is associated with fewer adverse events.

## Figures and Tables

**Table 1:** Main JAK-STAT pathways inhibited by use of JAKis in psoriasis [48,53,54,55,56,57,58,59,60,61].

Cytokines	Signaling Pathway		Effect upon Inhibition with JAKis
IL-12, -23	JAK2/ TYK2	STAT3, STAT4	inhibition if the IL-23/IL-17 axis, inhibition of Th1 differentiation, decreased IFN-γ production
IL-2, -4, -7, -9, -15, -21 (common γ-chain)	JAK1/ JAK3	STAT1, STAT3, STAT5, STAT6	inhibition of Th, cT, and NK-cell activity
IL-22	JAK1/TYK2	STAT3	normalization of keratinocyte differentiation and barrier function, reduction of antimicrobial peptide secretion by keratinocytes
IFN-γ	JAK1/ JAK2	STAT1	inhibition of Th1 activity, inhibition of T cell migration, inhibition of keratinocyte proliferation
IFN-α, -β	JAK1/ TYK2	STAT1, STAT2, STAT3	inhibition of T-cell mediated inflammation
EPO. TPO, GM-CSF	JAK2/ JAK2	STAT5	decrease in hemoglobin, lymphocyte, and neutrophil count

Summary of the JAK and STAT subtypes associated with the main cytokine receptors that are responsible for JAKi efficacy and associated side effects in the treatment of psoriasis. EPO—erythropoietin; TPO—thrombopoietin; GM-CSF—granulocyte-monocyte stimulating factor; Th—T-helper cells; cT cell—cytotoxic T cell; NK cell—natural killer cell.

**Table 2:** Summary of phase 2 and 3 RCTs of tofacitinib in psoriasis and psoriatic arthritis.

Tofacitinib								
Trial	Population—Diagnosis and No.	Type of Study	Trial Arms	Primary Endpoint	Results		Any Adverse Event	Serious Adverse Events
NCT00678210 Papp et al., 2012 [72]	Pso 197	phase 2	T2 T5 T10 placebo	%PASI75 at week 12	25% 40.8% 66.7% 2%		551% 57.1% 61.2% 60%	4.1% 2% 0 0
NCT01186744 Bissonnette et al., 2015 [75]	Pso 666	phase 3	Treatment period:					
			T5 T10	%PASI75 and PGA0/1 at week 24	33.5% 55.2%		61.9% 69.9%	1.8% 3.3%
			Treatment-withdrawal period:					
			T5 Placebo for T5 T10 Placebo for T10	% maintaining PASI75 at week 40	56.2% 23.3% 62.3% 26.1%		54.8% 50% 62.2% 42.9%	
				% maintaining PGA0/1 at week 40	49.9% 22.9% 63.9% 18%			
			Re-treatment period:					
			T5 T5 from placebo T10 T10 from placebo	% who relapsed and regained PASI75 at week 56	T5 from placebo	36.8%	29.6% 507% 40.5% 53.3%	3.7% 4% 2.4% 1.7%
					T10 from placebo	61%		
				% who relapsed and regained PGA0/1 at week 56	T5 from placebo	44.8%		
					T10 from placebo	57.1%		
NCT01241591 (OPT Compare) Bachelez et al., 2015 [76]	Pso 1101	phase 3	T5 T10 Etanercept Placebo	%PASI75	39.5% 63.6% 58.8% 5.6%		55% 60% 57% 51%	2% 2% 2% 2%
				%PGA score of 0 or 1 at week 12	47.1% 68.2% 66.3% 15%			
NCT01276639 (OPT Pivotal 1) Papp et al., 2015 [77]	Pso 901	phase 3	T5 T10 placebo	%PASI75 at week 16	39.9% 59.2% 6.2%		51% 61.1% 50.3%	2.2% 2.8% 2.8%
				%PGA 0/1 at week 16	41.9% 59.2% 9%			
NCT01309737 (OPT Pivotal 2) Papp et al., 2015 [77]	Pso 960	phase 3	T5 T10 placebo	%PASI75 at week 16	46% 59.6% 11.4%		55.8% 55.6% 47.4%	2.9% 1.3% 1%
				%PGA 0/1 at week 16	46% 59.1% 10.9%			
NCT01815424 Zhang et al., 2017 [78]	Pso 266	phase 3	T5 T10 Placebo	%PASI75 at week 16	54.6% 81.1% 12.5%		64.8% 67.8% 48.9%	2.3% 0 0
				% PGA 0/1 at week 16	52.3% 75.6% 19.3%			
NCT01882439 (OPAL Beyond) Gladman et al., 2017 [79]	PsA 395	phase 3	T5 T10 placebo	%ACR20 at 3 months	50% 47% 24%		55% 53% 44%	1% 2% 2%
				change in HAQ-DI at 3 months	−0.39 −0.35 −0.14			
NCT01877668 (OPAL Broaden) Mease et al., 2017 [80]	PsA 422	phase 3	T5 T10 adalimumab placebo	%ACR20 at 3 months	50% 61% 52% 33%		39% 45% 46% 35%	3% 1% 1% 1%
				change in HAQ-DI at 3 months	−0.35 −0.40 −0.38 −0.18			
NCT03486457 Leng et al., 2022 [81]	PsA 204	phase 3	T5 placebo	%ACR50 at 3 months	38.2% 5.9%		68.4% 75%	0 4.4%

Pso—moderate-to-severe plaque psoriasis; PsA—psoriatic arthritis; T2—tofacitinib 2 mg twice daily; T5—tofacitinib 5 mg twice daily; T10—tofacitinib 10 mg twice daily; %PASI75—percent of patients achieving a reduction of 75% of their baseline Psoriasis Area Severity Index score; %PGA 0/1—percent of patients achieving a Physician Global Assessment score of 0 “clear” or 1 “almost clear”; %ACR20/50—percent of patients achieving a 20/50% reduction in their baseline American College of Rheumatology response criteria; HAQ-DI—Health Assessment Questionnaire-Disability Index.

**Table 3:** Summary of phase 2 and 3 RCTs of deucravacitinib in psoriasis and psoriatic arthritis.

Deucravacitinib							
Trial	Population—Diagnosis and No.	Type of Study	Trial Arms	Primary Endpoint	Results	Any Adverse Event	Serious Adverse Events
NCT02931838 Papp et al., 2018 [102]	Pso 267	Phase 2	deucravacitinib 3 mg/2 d deucravacitinib 3 mg/d deucravacitinib 3 mg × 2/d deucravacitinib 6 mg × 2/d deucravacitinib 12 mg/d placebo	%PASI75 at week 12	9% 39% 69% 67% 75% 7%	59% 55% 64% 80% 77% 51%	2% 2% 2% 0 0 2%
NCT03624127 (POETYK PSO-1) Armstrong et al., 2023 [103]	Pso 666	phase 3	deucravacitinib 6 mg apremilast 30 mg placebo	%PASI75 at week 16 for deucravacitinib vs. placebo	58.4% vs. 12.7%	53% 55.4% 42.4%	2.1% 2.4% 5.5%
				%PGA 0/1 at week 16 for deucravacitinib vs. placebo	53.6% vs. 7.2%		
NCT03611751 (POETYK PSO-2) Strober et al., 2022 [104]	Pso 885	Phase 3	deucravacitinib 6 mg apremilast 30 mg placebo	%PASI75 at week 16 for deucravacitinib vs. placebo	53.0% vs. 9.4%	57.5% 59.1% 54.3%	1.6% 0.4% 1.2%
				%PGA 0/1 at week 16 for deucravacitinib vs. placebo	49.5% vs. 8.6%		
NCT03881059 Mease et al., 2022 [105]	PsA 203	Phase 2	deucravacitinib 6 mg deucravacitinib 12 mg placebo	% ACR20 at Week 16	62.7% 52.9% 31.8%	65.7% 65.7% 42.4%	0 0 1.5%

Pso—moderate-to-severe plaque psoriasis; PsA—psoriatic arthritis; %PASI75—percent of patients achieving a reduction of 75% of their baseline Psoriasis Area Severity Index score; %PGA 0/1—percent of patients achieving a Physician Global Assessment score of 0 “clear” or 1 “almost clear”; %ACR20—percent of patients achieving a 20% reduction in their baseline American College of Rheumatology response criteria.

**Table 4:** Summary of phase 2 and 3 RCTs of upadacitinib in psoriasis and psoriatic arthritis.

Upadacitinib							
Trial	Population—Diagnosis and No.	Type of Study	Trial Arms	Primary Endpoint	Results	Any Adverse Event	Serious Adverse Events
NCT03104400 (SELECT-PsA 1) McInnes et al., 2021 [108]	PsA 1704	phase 3	U15 U30 Placebo Adalimumab	%ACR20 at week 12	70.6% 78.5% 36.2% 65.0%	66.9% 72.3% 59.6% 64.8%	3.3% 6.1% 3.1% 3.7%
NCT03104374 (SELECT-PsA 2) Mease et al., 2020 [109]	PsA	phase 3	U15 U30 Placebo Adalimumab	%ACR20 at week 12	56.9% 63.8% 24.1%	64% 78% 65.6%	5.7% 8.3% 1.9%

PsA—psoriatic arthritis; U15—upadacitinib 15 mg daily; U30—upadacitinib 30 mg daily; %ACR20—percent of patients achieving a 20% reduction in their baseline American College of Rheumatology response criteria.

## Data Availability

Not applicable.
